# Attention-Deficit/Hyperactivity Disorder and Lifestyle-Related Behaviors in Children

**DOI:** 10.1371/journal.pone.0163434

**Published:** 2016-09-22

**Authors:** Lian Tong, Xu Xiong, Hui Tan

**Affiliations:** 1 Department of Maternal, Child and Adolescent Health, School of Public Health, Key Laboratory of Public Health Safety, Fudan University, Shanghai, China; 2 School of Public Health and Tropical Medicine, Tulane University, New Orleans, United States of America; Hospital Universitari i Politecnic La Fe, SPAIN

## Abstract

Attention-deficit/hyperactivity disorder (ADHD) has been associated with obesity in children. Lifestyle-related behaviors (external eating, screen time and physical inactivity) are well known to be associated with increased risk of obesity, but their associations with ADHD are unclear. The objectives of this study were to clarify the associations between ADHD symptoms in children and their associated lifestyle. A cross sectional study was carried out with a total of 785 primary students aged 9 to 13 years old and their parents were recruited by stratified random sampling from primary schools of China. The Cochran-Mantel-Haenszel (CMH) test was used to examine the relationships between ADHD symptoms and health related behaviors. We found that children with ADHD symptoms were likely to spend more time using a computer during school days; they were also more likely to eat while using a computer. These children were also more likely to eat while seated in a car, using a smart phone, using a computer at bedtime, and snacking before going to sleep than children without ADHD symptoms. An increased risk of obesity in children with ADHD symptoms was associated with the overuse of electronic devices, eating while using electronic devices, and delaying bedtimes to snack and use electronic devices.

## Introduction

Attention-Deficit/Hyperactivity Disorder (ADHD) is one of the most common childhood psychiatric disorders, affecting 5–10% of school-aged children worldwide [1, 2]. ADHD is defined by a persistent and age-inappropriate pattern of inattention, hyperactivity-impulsivity, or both [3]. It is well known that ADHD is associated with psychiatric and developmental disorders such as Oppositional Defiant Disorder, Conduct Disorder, Anxiety Disorders, Depressive Disorders, and Speech and Learning Disorders [4]. A possible comorbidity between ADHD and obesity has been suggested by recent studies [5–7]. Research indicates that individuals with ADHD may have an elevated risk for obesity in adults [8, 9] and youths [10–13]. Several large population studies have also observed an association between obesity and ADHD in the United States and China [6, 7, 14].

A number of potential mechanisms, some physiological and behavioral, have been hypothesized to explain the associations between lifestyle behaviors and obesity in people with ADHD. From a behavioral perspective, population survey data have suggested that ADHD is individually associated with lifestyle-related behaviors such as eating behaviors, screen time, and physical activity [15, 16]. It has been suggested that Children with ADHD were almost twice as likely to have fewer healthy behaviors, such as more artificially sweetened juice intake and higher screen time, even after adjustment for age, gender, household income, and other comorbid psychiatric disorders [17]. Children with ADHD displayed more disruptive patterns of eating behaviors and exhibited markedly diminished adherence to a traditional breakfast, lunch, and dinner schedule, which was linked to a significantly higher frequency of irregular eating times [18, 19]. It was said that deficits in attention and other executive functions in ADHD patients,might cause difficulties in adhering to a regular eating pattern, thus favoring external eating behaviors. Therefore, they may forget about eating when they are engaged in interesting activities and become more likely to eat when less stimulated, at which point they may be very hungry [20].

Additionally, high screen time exposure might increase the risk of obesity in both of normal children and children with ADHD. Previous studies examined the association between screen time and ADHD, but the results are contradictory [21]. A longitudinal study assessed the effects of early television exposure (at ages 1 and 3 years) on attention problems at age 7 in children. The results showed that an increase of one standard deviation the number of hours of television children watched at age 1 predicted a 28% increase in the probability of those children having attention problems at age seven [22]. Recent study showed that television exposure appears to be independently associated with ADHD symptoms in children [15, 16]. However, other two prospective studies found no significant associations between hours of watching TV and ADHD [23, 24]. Even though the findings in previous studies are contradictory, most researchers still emphasize the role of electronic devices in predisposing children to gain weight. The relationship between ADHD and screen time in ADHD children still needs to be examined further.

In addition to external eating and extended screen time, physical inactivity is another factor predictive of child obesity. Understandably, people have assumed that children with ADHD would lose weight easily due to a high level of physical activity, since hyperactivity would seem to increase daily energy expenditure. However, studies showed that children with ADHD were involved in few physical activities. A study found that children with ADHD, regardless of medication status and gender, are less likely to participate in vigorous physical activity and organized sports compared to those without ADHD [12]. Previous studies on movement skills and motor performance in ADHD children provide some insight into the underlying factors for the observed low physical activity in this population. For example, children with ADHD exhibit low gross motor performance, physical fitness, and delayed motor development [25, 26]. A recent qualitative study found that children with ADHD had only superficial knowledge about movement skills, paid little attention to specific details, and entertained negative feelings about physical activity [27].

Furthermore, children whose free time occurs prior to bedtime are at increased risk of excess food intake and screen-based sedentary behavior. An evening offering few chances for physical activity and relatively more opportunities for television viewing and other sedentary behaviors is known to be associated with increased snacking behavior [28] and exposure to advertisements for energy-dense foods in young people [29]. Few studies have examined the relationship between bedtime activities and ADHD. To examine the relationship among ADHD and external eating, screen time, and bedtime activities, particularly on the population level, we hypothesized that children with ADHD symptoms are more likely to use electric devices and eat more when using electric devices, as well as have few physical activities than the children without ADHD symptoms, especially in bedtime.

In the light of contradictory findings above and high relevance of lifestyle-related behaviors, it suggested that these behaviors should be considered as a whole, especially for children with ADHD. Because children with ADHD usually have multiple psychological comorbidities [30], these raise more complex behaviors. However, the previous studies haven’t evaluated the potential for combined effects from multiple healthy lifestyle behaviors, such as co-occur of eating behavior and screen time. Few studies have explored the bedtime activities in children with ADHD which may more likely to expose children into external eating, screen time and limited physical activities.

## Methods

### Participants and Ethics statement

Three primary schools were recruited by stratified and cluster sampling from Shanghai, China. These three schools are locating in central urban or suburb areas of Shanghai and represent different socioeconomic levels. As Table 1 showed that, parents in School A have highest socioeconomic level, e.g. comparatively high education level, high household income and young parents. On the contrary, the parents in School C have lowest socioeconomic level in the current samples. Considering the literacy, each student in grade 3, 4 and 5 and their parents were recruited. The age of the students is ranged from 9 to 13 years old (Mean = 10.6, SD = 1.1). A total of 810 dyads questionnaires were distributed and 785 dyads were collected. Twenty-five parent’s questionnaires were missing, since parents are not at home. The students’ questionnaires were requested to fill at self-study class and collected one hour later. Parents’ questionnaires were brought back to their parents and collected by teachers a week later. Prior to the survey, the informed consent forms were signed by parents if they agreed their children to attend to the survey. The content and procedure of this study were approved by the medical ethics committee of Fudan University. The corresponding author confirms that this study was performed in accordance with the approved social experiments guidelines and regulations.

**Table 1:** The variance in the demographic information of participated families in three elementary schools.

	School A	School B	School C	χ^2^_MH_
	N (%)	N (%)	N (%)	
Father's education level				
Illiteracy/Elementary school	6(1.8)	9(3.4)	13(7.2)	102.2****
Middle school	98(30.1)	82(31.4)	106(59.2)	
Senior school	71(21.8)	87(33.3)	47(26.3)	
Undergraduate college	135(41.5)	78(29.9)	13(7.3)	
Graduated college	15(4.6)	5(1.9)	0(0)	
Mother's education level				
Illiteracy/Elementary school	17(5.3)	21(7.9)	53(29.8)	146.6****
Middle school	94(29.4)	86(32.5)	93(52.3)	
Senior school	80(25.0)	78(29.4)	25(14.0)	
Undergraduate college	127(39.7)	77(29.1)	7(3.9)	
Graduated college	2(0.6)	3(1.1)	0(0)	
Annual household income (US$)				
≤3000	9(2.9)	16(6.3)	25(15.1)	71.3****
3000–6000	30(9.7)	34(13.4)	22(13.2)	
6000–9000	52(16.9)	51(20.2)	39(23.5)	
9000–12000	38(12.3)	29(11.5)	38(22.9)	
12000–15000	63(20.5)	38(15.0)	26(15.7)	
≥15000	116(37.7)	85(33.6)	16(9.6)	
Father's age				
<30	5(1.6)	2(0.7)	8(4.5)	46.4****
30–40	256(80.5)	154(58.8)	112(62.9)	
40–50	54(16.9)	97(37.0)	54(30.3)	
50–60	3(1.0)	9(3.5)	4(2.2)	
Mother's age				
<30	6(1.9)	6(2.3)	13(7.4)	43.5****
30–40	288(90.6)	210(79.5)	120(67.8)	
40–50	23(7.2)	46(17.4)	42(23.7)	
50–60	1(0.3)	2(0.8)	2(1.1)	
Single child				
Yes	239(73.5)	160(59.3)	55(30.4)	
No	86(26.5)	110(40.7)	126(69.6)	
ADHD symptoms				1.0
ADHD	247(90.8)	294(89.4)	162(88.0)	
No ADHD	25(9.2)	35(10.6)	22(12.0)	
Total	329(41.9)	272(34.6)	184(23.4)	

**** p <0 .0001

### Measures

#### ADHD symptoms

ADHD symptoms were assessed by the parent-report version of ADHD Rating scale-IV (ADHDRS-IV) [31]. The ADHDRS-IV is an 18-item ADHD assessment scale, which consists of two subscales, inattention and hyperactivity-impulsivity, each containing nine items. Each item is mapped onto one of the 18 DSM-IV symptoms of ADHD. Parents were required to rate the frequency of each of the ADHD symptoms occurring over the past six months on a five-point Likert scale with 0 for never or rarely, 1 for sometimes, 2 for often, and 3 for very often. The sum of all the scores on the 18 items results in a total score. The reliability and validity of the home version of ADHDRS-IV was verified in a sample of Chinese children aged 6–17 years [32]. Cronbach’s alpha of ADHDRS-IV for the present sample was .92. A cut-off point of 26 was used to define children with ADHD symptoms or not [32].

### Lifestyle-related behavioral variables

**External Eating behaviors:** External eating in this study was defined as eating accompanied with sedentary behavior, such as using electronic devices, doing homework, and snacking before bedtime. Because there is no validated questionnaire to measure the eating behaviors examined in this study, the self-designed questions were used. The option is a 4-point Likert response scale to evaluate how often the behavior occurs (i.e., never/seldom, sometimes, often, and always) which referred to 6-point Likert response scale of Child Eating Behaviour Questionnaire (CEBQ) [33]. Child participants were asked to rate “how often do you eat when you are doing the following things (1) watching TV; (2) using the computer; (3) walking, and (4) sitting in the car. For each item, the respondent indicates on a 4-point Likert response scale how often the behavior occurs (i.e., never/seldom, sometimes, often, and always).

**Screen time:** The participants were asked, “How much time did you spend using the following electric devices after school: (1) computer, (2) smart phone, (3) television.” The response options included: less than 30 minutes, 30–60 minutes, 1–2 hours, 2–3 hours, and more than 3 hours. This measure was based on a scale originally developed by Robinson [34]. Children are exposed to different electric devices at different times. For example, they are more likely to watch television on weekends or to use smart phones during the school day. The combined behaviors might be different in school and at home. Thus, the each item was analyzed separately, rather than as a sum of sedentary screen time activities.

**Bedtime activities:** Bedtime activities are defined here as the activities occurring after dinner and before bedtime, including eating, using electronic devices, and physical activities. For example, participating children were asked to report how many times they ate at bedtime in a week. Each question was rated on a five-point scale ranging from never to 7 days.

**Physical Activity:** Child participants were asked to report their physical activity by Physical Activity Questionnaire for Older Children (PAQ-C). The PAQ-C was developed to assess general levels of physical activity throughout the elementary school year for students in grades 4 to 8 and approximately 8 to 14 years of age. The validation and reliability of PAQ-C had been confirmed in a previous study [35]. The PAQ-C is a 10 items, self-administered, 7-day recall instrument. The mean of 9 items (excluding item 10) ranged from 1 to 5. A score of 1 indicates low physical activity, whereas a score of 5 indicates high physical activity. The internal consistency of PAQ-C for the current sample was acceptable for both females (α = 0.83) and males (α = 0.80).

**Sleep environment:** Parents were asked, “Are any of these electronic devices in your child’s bedroom?” Multiple options can be selected, such as televisions, video game systems, computers, the Internet, or none. The total number of electronic devices located in the child’s bedroom was calculated for analysis; that total ranged from 0 to 4. Additional survey items used to capture demographic information were completed by the children’s parents. One question asked of parents was, “Who shares a bedroom with your child?” Four options were available for this question: an individual bedroom, a room shared with siblings, a room shared with parents, or a room shared with other. The raw data was shown in S1 Data.

### Statistical analysis

The data was analyzed by Statistic Analysis System (SAS) 9.3 (Institute Inc., Cary, NC, USA). A Chi-square test was used to investigate the relationship between ADHD symptoms, demographic information, and sleep environment. The Cochran-Mantel-Haenszel (CMH) test was used to examine the relationship between ADHD symptoms and health-related behaviors. CMH was often used to examine a linear association between two variables that lie on an ordinal (or interval) scale [36]. A parameter of nonzero correlation (χ^2^_MH_) is reported in this study, since both variables are ordinal values. In addition, logistical regression analysis was carried out to examine the associations between ADHD symptoms and lifestyle-related behaviors, e.g. screen time, external eating behaviors and bedtime activities. For each behavior, a total score was summed up with a series of variables.

## Results

Table 2 indicated the proportion of ADHD symptomatic and non-symptomatic children. The results showed that 82 (10.5%) children had ADHD symptoms, with boys (n = 59, 7.5%) more frequently identified than girls (n = 23, 2.9%). Over half of the participating children shared a bedroom with others (n = 397, 50.6%). More children in the ADHD symptomatic group shared a bedroom others than the children in the non-symptomatic group (*χ*^*2*^ *=* 7.8840, p < .0001), especially in when the child shared a bedroom with his or her parents (n = 34, 42.5%). The proportion of single children was similar in the ADHD symptomatic group and the non-symptomatic group. There was no difference in annual household income (χ^2^ = 1.0800, p>.05) between children in the ADHD symptomatic group and the non-symptomatic group. In 256 bedrooms (29.6%), there were computers, and in 220 (28.1%) of the bedrooms were Internet-accessible computers. Televisions were found in 404 (51.5%) of the bedrooms, but only 24 (3.1%) bedrooms had video game consoles. There were 439 (55.9%) bedrooms with two or more than two devices.

**Table 2:** Differences between children with and without ADHD symptoms.

	Total, N (%)	ADHD, N (%)	No ADHD, N (%)	χ^2^
Gender				
Boys	409 (52.1)	59 (7.5)	350 (44.6)	14.5***
Girls	376 (47.9)	23 (2.9)	353 (45.0)	
Grade				
Three	268 (34.1)	34 (4.3)	234 (29.8)	2.2
Four	235 (29.9)	22 (2.8)	218 (27.1)	
Five	282 (35.9)	26 (3.3)	256 (32.6)	
Annual household income (US$; NA = 179, 22.8%)				
<3000	50 (6.9)	6 (0.8)	44 (6.1)	1.2
3000–6000	86 (11.9)	10 (1.4)	76 (10.5)	
6000–9000	142 (19.5)	16 (2.2)	126 (17.3)	
9000–12000	105 (14.5)	9 (1.3)	96 (13.2)	
12000–15000	127 (17.5)	13 (1.8)	114 (15.7)	
≥15000	196 (29.7)	20 (2.7)	176 (27.0)	
Mother's education (NA = 22, 2.8%)				
Illiteracy/Primary school	91 (11.9)	11 (1.4)	80 (10.5)	2.1
Middle school	273 (35.8)	31 (4.1)	242 (31.7)	
High school	183 (24.0)	18 (2.4)	165 (21.6)	
University	214 (26.6)	19 (2.5)	195 (25.1)	
Post college	5 (0.7)	0 (0)	5 (0.7)	
Father's education (NA = 20, 2.5%)				
Illiteracy/Primary school	28 (3.7)	3 (0.4)	25 (3.3)	3.2
Middle school	286 (37.3)	34 (4.4)	252 (32.9)	
High school	205 (26.9)	21 (2.8)	184 (24.1)	
University	226 (29.5)	17 (2.2)	209 (27.3)	
Post college	20 (2.6)	3 (0.4)	17 (2.2)	
Siblings				
Single child	454 (57.8)	48 (6.1)	406 (51.7)	0.0003
Non-single child	331 (42.2)	34 (4.3)	297 (37.8)	
Bedroom (NA = 12, 1.5%)				
Independent bedroom	388 (49.4)	28 (3.6)	360 (45.9)	10.4**
Share with siblings	66 (9.2)	8 (1.7)	58 (7.5)	
Share with parents	267 (34.5)	34 (4.4)	233 (30.1)	
Share with others	52 (6.7)	10 (1.3)	42 (5.4)	
Total	785 (100.0)	82 (10.5)	703 (89.6)	

NA means the number of missing data.

** p < 0.01,

*** p <0 .001

Table 3 indicates the association between ADHD symptoms and the screen-based sedentary behaviors of time, external eating, physical activity, and relation of behaviors to bedtime. Children with ADHD symptoms were more likely to use a computer on weekend (χ^2^_MH_ = 7.191, P<0.01). However, this difference was not statistically significant after adjusting for gender (χ^2^_MH_ = 3.7653, P>0.05). The results showed that boys spent more time than girls using the computer during school days (χ^2^_MH_ = 24.1219, P<0.05), on weekends (χ^2^_MH_ = 30.4438, P<0.001), and at bedtime (χ^2^_MH_ = 13.9939, P<0.001). Boys spent more time than girls watching TV on school days (χ^2^_MH_ = 5.7639, P<0.05), on weekends (χ^2^_MH_ = 5.2286, P<0.05), and at bedtime (χ^2^_MH_ = 8.9668, P<0.01). They also used smart phones more frequently on weekends than girls (χ^2^_MH_ = 19.6455, P<0.001).

**Table 3:** Associations between ADHD and screen time, external eating, bedtime activities and physical activity.

	N (%)	N (%)	N (%)	N (%)	N (%)	χ^2^_MH_^a^	χ^2^_MH_^b^
The time spend in using computer per day on school days							
	≤30mins	30-60mins	1–2 hours	2–3 hours	≥3 hours	7.191**	3.7653
No ADHD	459 (71.7)	93 (14.5)	58 (9.1)	12 (1.9)	18 (2.8)		
ADHD	44 (62.9)	10 (14.3)	7 (10.0)	2 (2.9)	7 (10.0)		
**Combined eating behaviors**							
1. Eating while using computer							
	never	sometimes	often	very often		10.332**	8.9416**
No ADHD	256 (37.2)	319 (46.3)	79 (11.5)	35 (5.1)	—		
ADHD	21 (26.9)	33 (42.3)	14 (18.0)	10 (12.8)	—		
2. Eating in the car							
No ADHD	289 (42.4)	326 (47.9)	56 (8.2)	10 (1.5)	—	9.4031**	8.5249**
ADHD	25 (32.5)	36 (46.8)	11 (14.3)	5 (6.5)	—		
**Bedtime activities (How many times per week)**							
	never	1–2 times	3–4 times	5–6 times	7 times		
1. Eating snacks							
No ADHD	376 (54.8)	245 (35.7)	43 (6.3)	8 (1.2)	14 (2.0)	9.4942**	8.5285**
ADHD	34 (44.2)	30 (39.0)	5 (6.5)	(1.3)	7 (9.1)		
2. Drinking soft drinks							
No ADHD	293 (43.0)	314 (46.0)	52 (7.6)	20 (2.9)	3 (0.4)	7.5222**	6.0182*
ADHD	32 (41.6)	33 (42.9)	6(7.8)	2 (2.6)	4 (5.2)		
3. Using smart phone							
No ADHD	410 (60.0)	194 (28.4)	55 (8.0)	17 (2.5)	8 (1.2)	6.5998*	5.2357*
ADHD	38 (48.7)	27 (34.6)	7 (9.0)	0	6 (7.7)		
4. Using computer							
No ADHD	420 (61.2)	192 (28.0)	40 (5.8)	12 (1.8)	22 (3.2)	6.6733**	4.0351*
ADHD	34 (44.2)	30 (39.0)	7 (9.1)	1 (1.3)	5 (6.5)		
5. Physical activity level							
No ADHD	264 (38.7)	199 (26.2)	132 (29.2)	49 (7.2)	38 (5.6)	1.7082	1.7104
ADHD	35 (45.4)	22 (28.6)	14 (18.2)	2 (2.6)	4 (5.2)		
Physical activity level in the last week							
No ADHD	12 (2.0)	225 (38.0)	302 (51.0)	48 (8.1)	5 (0.8)	1.3772	1.6422
ADHD	2 (2.9)	32 (47.1)	27 (39.7)	7 (10.3)	0		
Number of electronic devices in the bedroom of children							
	0	1	2	3	4		
No ADHD	196 (27.9)	312 (44.4)	108 (15.4)	77 (11.0)	10 (1.4)	6.6353*	6.9314**
ADHD	16 (19.5)	34 (41.5)	15 (18.3)	14 (17.1)	3 (3.7)		

* p < 0.05,

** p < 0.01

^a^. Cochran-Mantel-Haenszel (CMH) test without gender adjustment;

^b^. CMH test with gender adjustment.

As mentioned above, there was a gender difference in the total time children spent using electronic devices. However, there was no gender difference in the association between eating behaviors combined with screen time and ADHD symptoms. Specifically, after adjusting for gender, children with ADHD symptoms were more likely to eat when they are using a computer (χ^2^_MH_ = 10.332, P<0.01) or sitting in a car (χ^2^_MH_ = 9.4031, P<0.01). In addition, children with ADHD symptoms tended to eat snacks (χ^2^_MH_ = 9.4942, P<0.01), consume soft drinks (χ^2^_MH_ = 7.5222, P<0.01), and use smart phones (χ^2^_MH_ = 6.5998, P<0.05) and computers (χ^2^_MH_ = 6.6733, P<0.01) at bedtime more frequently than children without ADHD symptoms. Moreover, children with more ADHD symptoms had more electronic products in their bedrooms after adjusting for gender (χ^2^_MH_ = 6.9314, P<0.01). In the present study, the physical activity level was not found to be associated with ADHD symptoms either at bedtime (χ^2^_MH_ = 1.710, P>0.05) or during the previous week (χ^2^_MH_ = 1.6422, P>0.05). The mean activity score of PAQ-C was 2.65 (SD = 0.70) for girls and 2.70 (SD = 0.69) for boys.

The results of multiple regression analysis were shown in Table 4. It suggested that children with ADHD symptoms showed more combined eating behaviors (β = 0.04, P<0.01), bedtime activities (β = 0.05, P<0.05) and more electronic devices in bedroom (β = 0.01, P<0.01) after controlled for children’s gender, parents and children’s age, parents’ education level and annual household income. No significant association was found between ADHD symptoms and screen time after adjusted for the demographic information. However, children’s gender (β = -1.27, P<0.001), especially mother’s educational level (β = -1.27, P<0.001) and age (β = 1.26, P<0.00) were related to children’s screen time. Boys have longer screen time than girls. Mothers have higher educational level and younger, their children tend to have shorter screen time. Higher mother’s education level also predicted children’s less unhealthy bedtime activities (β = 0.56, P<0.05).

**Table 4:** Multiple regression analysis to explore the associations between ADHD and lifestyle-related behaviors.

	Screen time / β	Combined eating / β	Bedtime activities / β	Physical activity / β	Electronic devices in bedroom/ β
ADHD	0.04	0.04**	0.05*	-0.01	0.01**
Children's gender	-1.27***	-0.22	-0.84*	-0.07	0.03
Children's age	0.14	-0.08	0.06	-0.01	0.01
Father's education level	0.23	0.22	0.14	-0.03	-0.02
Mother's education level	-0.74**	-0.33*	-0.56*	0.01	-0.06
Father's age	-0.49	0.05	0.71	-0.01	0.07
Mother's age	1.26*	0.06	0.70	0.17	0.09
Annual household income	0.12	-0.05	-0.01	0.02	0.02

* P < 0.05,

** p < 0.01,

*** p < 0.001;

The dependent variables are each lifestyle-related behaviors. The independent variables are ADHD total score, and all demographic information is adjusted factor.

## Discussion

Previous literature has examined lifestyle-related behaviors with ADHD separately, for example screen time and eating behaviors, but co-occur of lifestyle behaviors has not been addressed yet. Specifically, in this study, a unique finding in this study is that children with ADHD symptoms frequently combine eating with sedentary behaviors after controlled for parents’ education level and age, and children’s gender and age, e.g., snacking when using a computer or sitting in a car. A possible explanation is related to the characteristics and symptoms of ADHD; that children with ADHD have significantly greater impulse control deficits and loss of control over eating than children without ADHD [37]. The deficits in self-regulation and increased impulsivity may contribute to externally-cued eating, eating in the absence of hunger or binge eating, behaviors associated with obesity [14, 37]. Physiologically, dysfunction of the dopamine receptor gene DRD2 gives rise to a ‘reward deficiency syndrome’ that is associated with increased risk taking, substance abuse and eating pathology [38, 39]. A study indicated that ADHD can increase snack intake in Korean young children [40]. Hence, external eating may be a result of ADHD characteristics for these children with being overweight or obese as an outcome. Another reason might be of a high level of motoric impulsiveness in children with ADHD, which drives them do something else when they are sitting. A high level of motoric impulsiveness is often observed in patients with bulimia nervosa and binge eating behaviors [41, 42]. Compulsive eating may be a compensatory mechanism to help children with ADHD control the frustration associated with attention and organization difficulties.

Another finding in this study is that children with ADHD symptoms tended to eat snacks and drink sodas at bedtime more frequently than children without ADHD symptoms. These eating behaviors might be associated with watching television or using a computer, or they may occur independently. In either case, evening eating increases a child’s risk of becoming overweight [43]. Perhaps the evening hours offer little opportunity for outdoor activities and more time for sedentary behaviors, so a long period of free time before bedtime poses a challenge for children with ADHD. Staying at home at evening potentially increased the risk of co-occur of unhealthy behaviors. Furthermore, children with ADHD symptoms may have higher tendency to consume food as means of self-management since food can play a role similar to stimulant medication in reducing the distraction [44].

In addition, we found that children with more ADHD symptoms spent more time using computer on school days. However, the association was attenuated after adjusting for the gender of children. This finding seems reasonable because children with ADHD are predominantly boys. The conjecture has been confirmed by our finding in this study that boys have more screen time than girls. A previous study also suggested that male adolescents spent more time than girls watching TV and using a computer for leisure activities or both combined [45, 46]. Since daytime computer use for primary students in China is prohibited except for special activities, we speculate that children are more likely to use computer after school. We also found that boys and girls with ADHD symptoms spent more time using a computer and smartphone at bedtime than children without ADHD symptoms. Another interesting finding of the present study may support this explanation as well. We found that more electronic devices are accessible in the bedrooms of children with ADHD symptoms than for children without ADHD symptoms after adjusted for parents’ education level and household income. Children with ADHD symptoms are more likely to share a bedroom with others, especially with their parents. It might be because Chinese parents usually let their children sleep with them at night for safety, which may increase a child’s use of electronic devices at bedtime. Another reason is that parents are willing to restrict the unwanted behaviors of children with electronic devices, since restless children will become quiet when they are involved in video games or movies.

However, when taking the screen time in a week as a whole, the association between ADHD symptoms and screen time no longer existed after controlled for parents’ education level, age, and children’s age and gender. It turns out that mother’s education level and age have a significant association with children’s screen time. It might because well educated and young mothers may pay more attention to promote children’s healthy behavior. It’s well known that mother’s educational level is associated with children’s health and school achievement [47, 48]

Furthermore, it should be noted that increased screen time by itself does not decrease physical activity. This finding might seem counterintuitive given that many children with ADHD have symptoms of hyperactivity, suggestive of a high level of physical activity. However, our study demonstrated that children with ADHD symptoms had more screen-based sedentary behaviors but no difference in their physical activity level compared to children with and without ADHD either in the previous week or at bedtime. The findings are consistent a previous study that found screen-based sedentary behavior and leisure-time physical activities to be largely independent behaviors [49]. Physical activities have multiple benefits for children with ADHD, so high level of physical activities may hided children’ s ADHD symptoms [50, 51]. Another reason might be because children in China generally have lower level of physical activity than their counterparts in Canada by using the same assessment tool for physical activity regardless of the gender of children [52]. On a national level, negative associations between physical activity and screen-based sedentary behaviors are less likely to be found in countries with relatively low levels of physical activity [53].

In conclusion, we found that children with ADHD symptoms have more unhealthy lifestyles and behaviors, including external eating and bedtime eating. It’s worth noticing that these behaviors may co-occurred more frequently in children with ADHD. Particularly, eating behaviors often combined with screen time and other sedentary behaviors, or eating in bedtime. These behaviors might be associated with an increased risk of obesity. The strengths of this study were a comparatively large sample size, randomized sampling with good representation, study from a developing country in Asia, and interesting findings. It offered new specific evidences to support behavioral intervention and health management for children with ADHD symptoms. Meanwhile, the import role of mother should be taken into consideration in a family-based intervention program.

## Limitations

Firstly, this study uses a questionnaire and not interview-based assessments to assess ADHD symptoms. Physical activity was measured by self-reported questionnaire rather than accelerometers. Secondly, it is also a cross sectional study, limiting the ability to comment on causality. A longitudinal study should be carried out in the future to examine the causality between ADHD symptoms and unhealthy lifestyle-related behaviors, and the longitudinal health outcomes, especially for obesity.

## Supporting Information

S1 Data Lifestyle-related behaviors in children. (XLS)
